# Baking Optimization as a Strategy to Extend Shelf-Life through the Enhanced Quality and Bioactive Properties of Pulse-Based Snacks

**DOI:** 10.3390/molecules25163716

**Published:** 2020-08-14

**Authors:** Daniel Rico, Ana M. González-Paramás, Cristina Brezmes, Ana Belén Martín-Diana

**Affiliations:** 1 Subdirection of Research and Technology, Agro-Technological Institute of Castilla y León, Consejería de Agricultura y Ganadería, Finca de Zamadueñas, Ctra. Burgos km. 119, 47171 Valladolid, Spain; ricbarda@itacyl.es; 2 Polyphenol Group, Campus Miguel de Unamuno, University of Salamanca, 37007 Salamanca, Spain; paramas@usal.es (A.M.G.-P.); cristina@hotmail.com (C.B.)

**Keywords:** temperature, time, antioxidant, antihypertensive, baking, pulses, snacks

## Abstract

Food processing optimization can enhance the nutrient bioavailability, storage time, and stability of convenience foods. Baking is a heat and mass transfer process with a high impact on the shelf-life of the obtained product; a small variation in the parameters during baking can lead to significant changes in the end baked product, as it significantly affects the food nutrient profile and bioactive compounds. Response surface methodology (RSM) was used for mapping a response surface over a particular region of interest of baking conditions. The combined effect of the two factors (baking temperature and time) on the selected quality and bioactive parameters as dependent factors was evaluated in order to predict the optimal baking conditions which can facilitate the extended shelf-life of the product through maximizing the antioxidant bioactive properties. This design was used to develop models to predict the effect of the temperature and time baking profile and select those conditions where the quality and bioactive parameters reached a balance to obtain pulse snacks with a high quality, enhanced bioactive properties, and thus a longer shelf-life. Simultaneous optimization by the desirability function showed that a maximum temperature of 210 °C and a time of 14 min were the optimum conditions to produce a pulse-based snack with high antioxidant-antihypertensive activity and nutritional quality.

## 1. Introduction

Extending the shelf-life of food products is a challenging task, especially when the processing and storage conditions need to ensure optimal nutritional, organoleptic, and health-related properties. In the case of snacks which include pulses in their formulation, the processing has to comply with further needs, such as anti-nutrient elimination or increasing the protein digestibility.

The definition of a snack may be as simple as a food item that is consumed between meals, and therefore snacking has been a characteristic of the human diet ever since [1]. Nevertheless, defining a snack as healthy or not appears to be a more complex task [2]. An important attribute of snacks is convenience, and the widespread use of marketing strategies, such as those focused on high exposure (point of purchase display), may induce unhealthy impulsive purchases [3]. However, as consumer demand for healthy choices is increasing [4], it is important to increase the available range of snack products that, on one hand appeal to the consumer for their nutritional and health-related properties and, on the other, are commercially competitive through optimal processing conditions that ensure a long shelf-life, maintaining the nutritional and bioactive properties of the product.

The use of novel ingredients and formulations provides customers with new choices in their interest to combine snacks benefits with health and satiety and are indulgent. The consumption of cereal-based snacks has increased by 11% across the world [1] over the last five years. Cereal snacks are very popular and convenient, however usually these snacks contain high fat and high sugar content [5], and therefore are relatively nutrient poor. Pulses are an adequate method to increase the biological value of proteins in cereal-based products [6]. The health-promoting properties of pulses have been deeply investigated; their consumption has been associated with a wide range of health benefits, including a reduced risk of chronic diseases (diabetes, obesity, cardiovascular pathologies) and certain types of cancers. These beneficial effects have been associated with the presence of phenolic compounds, within other phytochemicals and peptides with antioxidant and antihypertensive bioactivities. Moreover, pulses are a good source of slowly digestible carbohydrate and fiber, effectively lowering the glycemic index (GI).

*Vicia narbonensis* (L.), known as Narbon bean or Narbon vetch, is a pulse grain grown mainly for forage and as a cover crop in dry areas, providing important benefits for the development of sustainable agriculture [7]. Due to its nutritive value, besides the fact that this crop is not usually used for human consumption, Narbon beans have been traditionally used for animal feeding as an interesting alternative to reduce the high dependence of soybean [8]. Del Pino-García et al. [9] reported results on the bioactive properties of *Vicia narbonensis* and demonstrated the need to select certain varieties to reduce the presence of the antinutrient g-glutamyl-S-ethenyl-cysteine (GEC), a sulfur-containing dipeptide [10,11,12]. Active breeding and research programs have been developed in recent years to reduce the GEC content in Narbon beans and provide information for establishing the limits of inclusion of this grain legume in feed formulations, which are set at 10–12.5%, although a GEC acceptance threshold for different species has not yet been defined [11,13]. In the context of human nutrition and within a varied and well-balanced diet, Narbon bean could be an excellent source of vegetable nutrients and bioactive components, although advances in the selection of the most suitable cultivars for this aim are required.

Shelf-life limiting factors and the quality loss of snacks are generally related to textural changes, which are affected by increased moisture content. Rancidity is another important quality loss factor in those snacks with a high fat content. According to Peryam [14] and Dethmers [15], shelf-life involves different aspects, such as formulation, processing, and storage conditions. In the case of baked snack products, parameters such as firmness and dryness are limiting parameters to preserve this type of product. Advances in bakery processing technologies, formulation, and ingredient innovation have significantly improved shelf-life extension; however, the incorporation of novel food matrices in snacks over the last few years has increased the demand to optimize.

Processing has an important impact on food nutrients. In the case of baked products, baking conditions, mostly time and temperature, significantly affect the final product. Baking brings about a number of physical and chemical changes [16]; these changes can be beneficial or detrimental, depending on the extent and type of treatment conditions. Baking is a complex process which involves many physical, chemical, and biochemical changes in food. In particular, during baking starch gelatinization occurs, affecting the palatability, digestibility, and softening of the raw starch matrix [17]. However, very little information is available regarding the temperature and time baking conditions required to obtain the optimal nutritional and/or antioxidant properties of baked products, particularly legume-containing snacks.

Baking processes may have an effect on the antioxidant content of food due to the antioxidant release, destruction, or creation of redox-active metabolites [18]. Antioxidant compounds, such as ascorbic acid and some carotenoids, are very sensitive to heat and storage. However, polyphenols have shown a certain stability when exposed to high temperatures [19]. In this regard, changes in these compounds may have important effects on the bioactive properties of the final product and also on its shelf-life, as they are related to oxidative processes.

The objective of this work was to optimize the time and temperature profile conditions of the baking process of a pulse snack to maximize the bioactivity and quality in order to obtain a snack with optimal shelf-life through the use of response surface methodology (RSM) and multiple linear regression (MLR).

## 2. Results and Discussion

Pulse-based snacks are gaining unprecedented popularity in the snack market due to the association of its consumption with the promotion of health [20]. The study was carried out to describe the effect of baking temperature and time in order to obtain optimal conditions for a pulse snack with appropriate quality and nutritional and bioactive properties during its shelf-life.

### 2.1. Proximal Analysis

The baking time-temperature profile did not produce any significant effect on the protein content (Table 1, Equation (1)). Most proteins undergo structural unfolding and/or aggregation when subjected to the baking process, which can produce an improvement in the protein digestibility and bioavailability; however, this process enhanced the lysine loss under severe conditions of temperature at low-moisture conditions and with the presence of reducing sugars [21]. In the case of fat content, the time and temperature did not produce any effect, similarly to that observed for protein (Table 1, Equation (2)).

(1) $$\begin{array}{c} \mathrm{Protein}=22.436-0.0377197\times X-0.640305\times Y+0.0000727379\times X^{2}\\ +0.00075\times X\times Y+0.0206098\times Y^{2} \end{array}$$

(2) $$\begin{array}{c} \mathrm{Fat}=13.2525-0.00395499\times X-1.92927\times Y-0.00000689627\times X^{2}\\ +0.00241667\times X\times Y+0.0616734\times Y^{2} \end{array}$$

Carbohydrate content was significantly affected (*p* ≤ 0.05) by temperature; carbohydrate content decreased with increasing temperature. A quadratic temperature effect was observed, finding the minimum values at 180 °C (Table 2, Equation (3)). However no significant (*p* ≥ 0.05) effect of time or interaction between temperature and time was observed. The dietary fibre showed a correlation with the results observed for carbohydrate, and the heat treatment during the baking process affected the dietary fibre (Table 2, Equation (4)).

(3) $$\begin{array}{c} \mathrm{Carbohydrates}=90.9625-0.209973\times X+0.872973\times Y+0.000506834\\ \times X^{2}+0.00183333\times X\times Y-0.0497722\times Y^{2} \end{array}$$

(4) $$\begin{array}{c} \mathrm{Fiber}=11.6684-0.032952\times X+0.119\times Y-0.0000392888\times X^{2}+0.005\times X\\ \times Y+0.039898\times Y^{2} \end{array}$$

The increase in the temperature probably led to a breakage of weak bonds between polysaccharide chains, resulting in a significant (*p* ≤ 0.05) linear increase in dietary fibre at high temperatures. The maximum values of fibre were observed at higher temperatures. A similar behavior, an increase in dietary fiber with longer baking times, was observed in other studies [22]. It also important to note that dietary fibre content can be associated with the formation of resistant starch fractions. The fast evaporation of water from the crust owing to its high surface temperature impairs the full gelatinization of the starch [23], which may result in a higher fraction of native (ungelatinized) resistant starch.

(5) $$\begin{array}{c} \mathrm{Ash}=5.38085-0.0154623\times X-0.100917\times Y+0.0000180667\times X^{2}\\ +0.000583333\times X\times Y+0.000196668\times Y^{2} \end{array}$$

**Figure 1:**
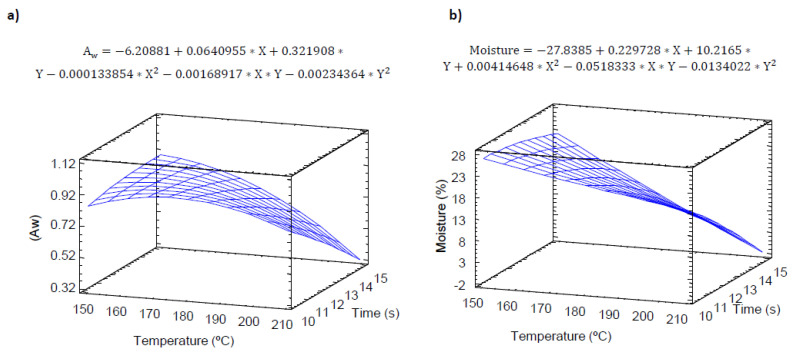
Response surface model of the effect of temperature and time on (**a**) a_w_ and (**b**) moisture.

It is well known that the textural properties and especially the crispness of dry foods is associated with water content, and the loss in shelf-life due to changes in these properties appears when the water activity or water content of the products rises above a critical value. For this reason, the water activity and humidity were monitored for the experimental design. Figure 1a shows the effect of baking (temperature and time) on the water activity of pulse snacks, expressed as the ratio between the vapor pressure of the food itself, when in a completely undisturbed balance with the surrounding air media, and the vapor pressure of distilled water under identical conditions.

R-squared indicated that the model explained 88.5% of the variability of a_w_ associated with the effect of time and temperature. According to Figure 1a, both temperature and time were significant (*p* ≤ 0.05). The increase in temperature and time significantly reduced (*p* ≤ 0.05) the a_w_ of the product, showing at higher baking times and temperatures lower aw values. Therefore, the water activity showed a negative exponential behavior, with the minimum a_w_ values at maximum baking temperatures (210 °C). This may be associated with a higher starch gelatinization at lower temperatures [23], increasing the water holding capacity. The lowest a_w_ value found (0.32) was below the recommended (0.57) to avoid any perceptible change in flavor for 24–52 days [24].

Moisture (Figure 1b) was also evaluated as a quality parameter in snacks. The results showed a significant linear temperature effect (*p* ≤ 0.05), reaching the lowest humidity values at longer baking times. Moisture content plays a significant role in the texture of snacks, as it is directly a key factor in their acceptance. According to Mazumder et al. [25], the moisture content in snacks should be below 10%. To reach these moisture values and assess the quality and shelf-life, the model showed the need to use temperatures of over 200 °C and times longer than 12 min.

### 2.2. Phenolic Content and Characterization

**Figure 2:**
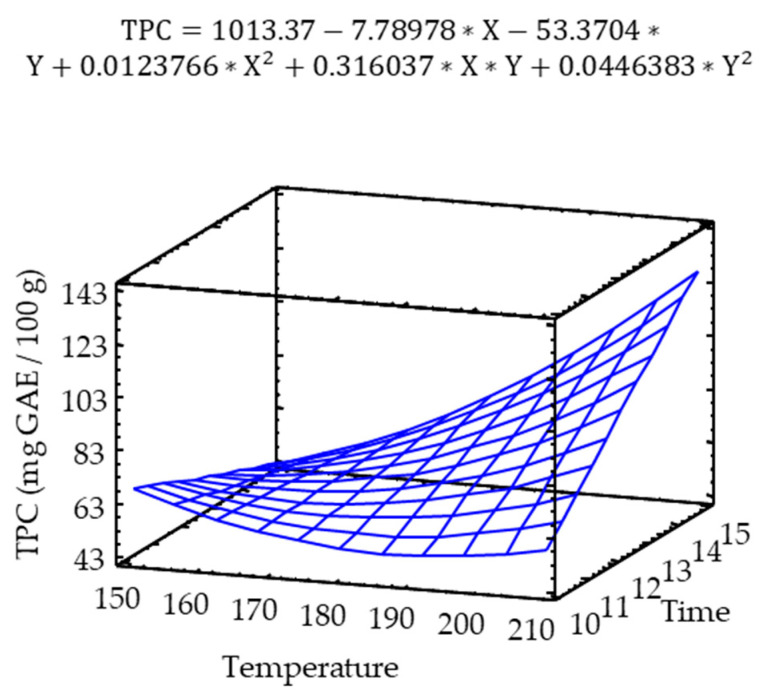
Response surface model of the effect of temperature and time on the TPC.

The total phenolic content of the pulse snacks was analyzed using the Folin-Ciocalteu method, and the results found were in the range of 90 to 290 µmol Gallic Acid Equivalent (GAE)/100 g. According to Figure 2 and the obtained model, there was a significant interaction effect of temperature and time on the observed Total Phenolic Content (TPC). The results showed a positive exponential TPC behavior, with the maximum values observed at high baking temperatures and times (210 °C, 14 min). The TPC was significantly affected by temperature and time (Supplementary Table S1 and Supplementary Figure S1).

These results agreed with those reported by Randhir et al. [26]. The changes in the total phenolic content caused by thermal processing could be associated with the breakage or complete modification of cellular components by heat.

Phenolic compounds can conjugate with sugars, fatty acids, or proteins [27]. Therefore, it can be suggested that the dissociation of the conjugated phenolic forms may have led to a certain polymerization of the phenolic constituents, which may be responsible for this increase in the observed TPC, an effect associated with a hydrolytic process caused by the processing.

It is also known that phenolic compounds accumulate in vacuoles [28], and thermal processing can release, during decomposition, more bound phenolic acids than those that are part of the cell or the membrane. However, it is important to bear in mind that the apparent increase in TPC might be due to the presence of melanoidins that form during the Maillard reaction, which react as reducing agents with the Folin Ciocalteau reagent [29].

**Figure 3:**
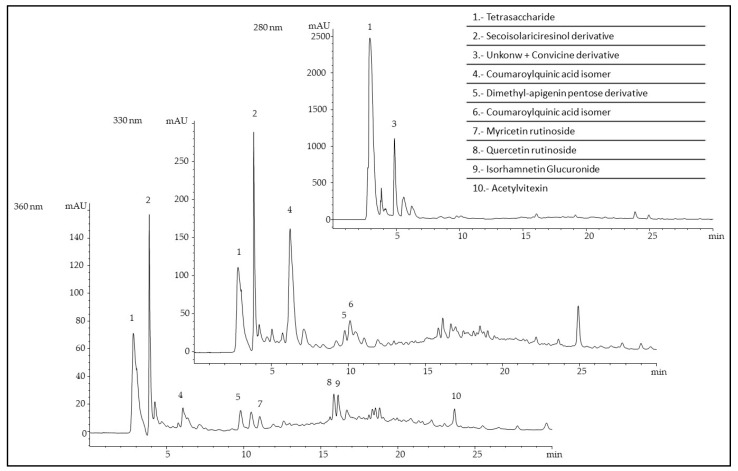
Chromatograms of the phenolic profile of the pulse *Vicia narbonensis* as detected at three wavelengths, 260, 280, and 330 nm.

Representative HPLC chromatograms registered at different wavelengths (280, 330, and 360 nm) are shown in Figure 3. Ten main compounds were detected in the methanolic extract of *V. narbonensis* flour, belonging to the families of lignans (one compound), hydroxycinnamic acids (two compounds), flavonols (three compounds), flavones (two compounds), oligosaccharides (one compound), and alkaloid glycosides (one compound).

The main peak at 280 nm (peak 1) was tentatively identified, based on the mass spectral data, as a tetrasaccharide, probably stachyose, which is recognized as a major storage and transport sugar in woody plants, cucurbits, and legumes [30].

The pyrimidine glycosides, vicine and convicine, are compounds present in the genus *Vicia*, including *Vicia narbonensis* [31]. In the present sample, a small amount of convicine has been detected, coeluting with an unknown compound (peak 3). Although this alkaloid glycoside is considered as an anti-nutritional factor, their concentration can be greatly reduced by hydrolysis or fermentation prior to baking [31,32].

Peak 2 has been tentatively identified as a secoisolariciresinol derivative, one of the lignans present in legumes. Lignans and isoflavones are considered as phytoestrogens and, although the epidemiological results are controversial, lignan-rich diets may be beneficial, particularly if consumed for health purposes. Experimental evidence in animals has shown clear anticarcinogenic effects of flaxseed or pure lignans in many types of cancer [33,34].

On the other hand, the presence of flavones is also considered a characteristic of legumes. In the analyzed sample, two apigenin derivatives (peaks 5 and 10) were detected. Numerous pharmacological activities, including anti-hypertensive, inflammatory, anti-toxicant, anti-cancer, etc., are attributed to apigenin [35]. The detected phenolic acids corresponded mainly to coumaroylquinic acid derivatives. The conjugates of coumaric acid have been extensively studied in recent years due to their bioactivities, including antioxidant and anti-inflammatory, and their effects against diabetes [36].

Although in lower amounts than hydroxycinnamic acids, flavonols have been also detected in the analyzed flour, corresponding to myricetin (peak 7) and quercetin (peaks 8 and 9) derivatives. Numerous authors have reported a strong antioxidant activity for quercetin, describing its capacity to maintain oxidative balance, manifested through its effect on glutathione (GSH), enzymatic activity, signal transduction pathways, and reactive oxygen species (ROS) [37]. On the other hand, quercetin was reported to interact with many molecular targets in the small intestine, pancreas, skeletal muscle, adipose tissue, and liver to control whole-body glucose homeostasis [38].

### 2.3. Total Antioxidant Capacity (TAC)

**Figure 4:**
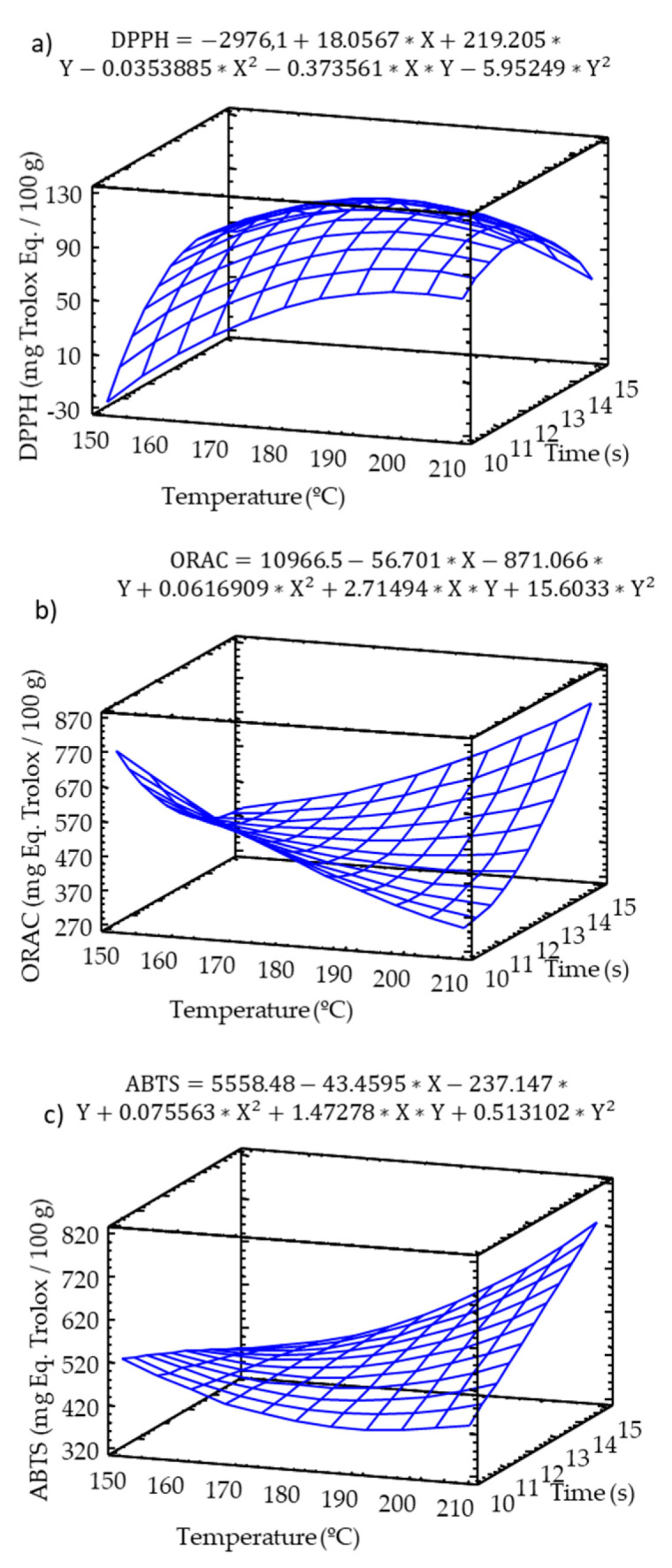
Response surface model of the effect of temperature and time on (**a**) DPPH, (**b**) ORAC, (**c**) ABTS.

The TAC of extracts was evaluated using the DPPH, ORAC, and ABTS methods, and the TAC of solid samples was evaluated through direct methods with DPPH and ABTS radicals. A significant (*p* ≤ 0.05) increase in the DPPH radical scavenging capacity was observed at higher temperature–time baking conditions (Figure 4a), although this effect was not significant. In the case of the peroxyl radical scavenging ability, the ORAC values (Figure 4b) showed a significant (*p* ≤ 0.05) interactive effect of the independent variables (temperature-time) on this antioxidant marker. Higher ORAC values were observed at either the mildest (low temperature and time) or most intense (high temperature and time) baking conditions.

The ABTS radical scavenging capacity was significantly modified by the baking conditions. An increase in temperature resulted in an increase in the ABTS values (Figure 4c). A significant interactive effect between the independent variables (temperature and time) was observed, with the highest ABTS scavenging capacity corresponding to most intense baking conditions (high temperature and time). The ABTS values showed a significant correlation with the TPC (R^2^ = 70.5%).

The results were in agreement with those of Vogrincic et al. [39], who attributed the increase in antioxidant activity during baking to the production of antioxidant compounds, partially due to the Maillard reaction products and glycosylation induced by thermal processing in phenolics, which may influence their antioxidant activity [40,41]. The presence of hydroxycinnamic acids and flavonols could respond to the high antioxidant activity of snacks within the melanoidins produced in the Maillard reactions.

The melanoidins produced in the Maillard reactions between proteins and sugars in real foods are mostly high molecular weight (HMW) compounds. Considering that the molecular weight of melanoidins produced is highly dependent on the heating intensity, it is possible that in the initial stages of baking, low molecular weight melanoidins (LMW) are more abundant melanoidins, which polymerize or cross-link to produce HMW melanoidins [42]. Del Castillo et al. [43] reported that the HMW melanoidins produced during bread baking showed a stronger peroxyl radical scavenging activity than the LMW melanoidins [44,45,46]. In this sense, the results observed for the ORAC assay may be explained by the peroxyl radical inhibitory activity of HMW melanoidins, which increases with higher temperature and time processing, as occurred with the increased ORAC values at the mentioned conditions. The different behaviors observed in the DPPH results, with the increasing antioxidant activity related to the temperature increase, could be attributed to differences in the solubility of melanonid compounds. The HMW melanoidins were poorly soluble, and DPPH showed a lower reactivity with these compounds compared to HMW.

**Figure 5:**
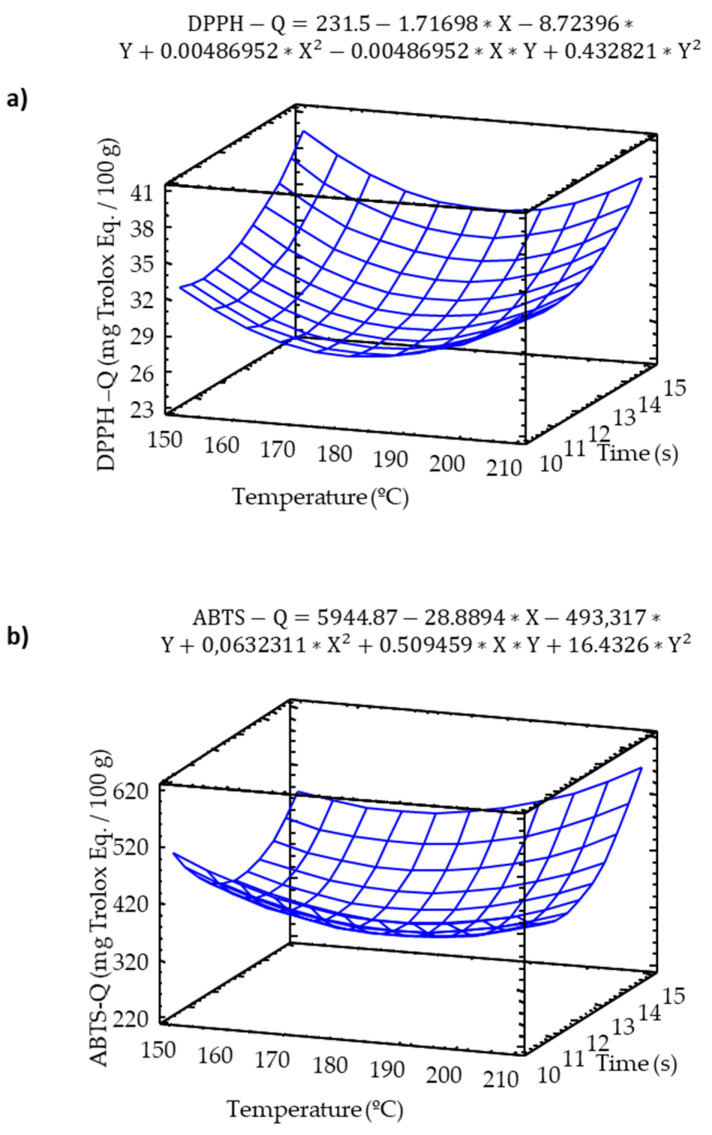
Response surface model of the effect of temperature and time on (**a**) DPPH-Q, (**b**) ABTS-Q.

An analysis of direct methods were completed in order to measure the contribution of the whole sample to the total antioxidant activity. Both Q-DPPH and Q-ABTS showed a slight increment in the antioxidant activity at high temperature and baking times (Figure 5).

### 2.4. Angiotensin Converting Enzyme (ACE)

**Figure 6:**
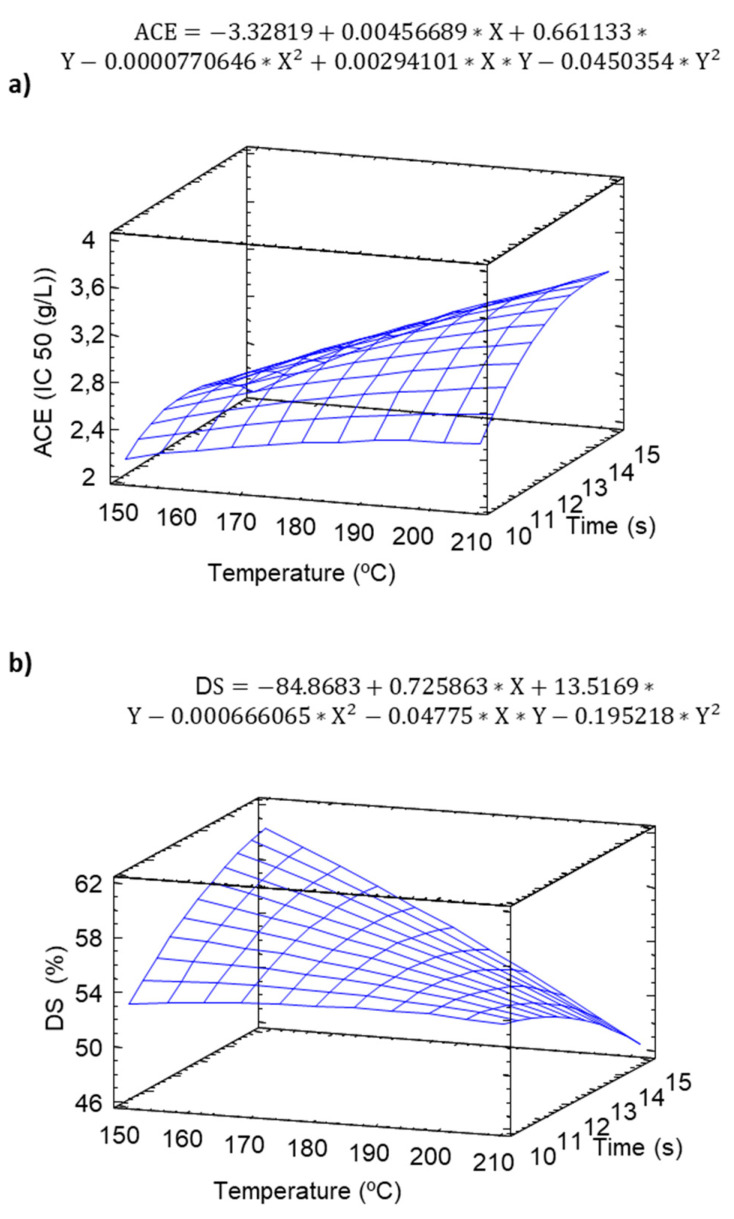
Response surface model of the effect of temperature and time on (**a**) angiotensin converting enzyme (ACE) and (**b**) Digestible Starch (DS) of pulse snacks.

ACE catalyzes the hydrolysis of angiotensin-I peptide and plays an important role in the production of angiotensin-II vasopressor peptide in blood [47]. ACE showed a significant increasing linear behavior, reaching maximum values with maximum baking temperatures (210 °C) and long baking times (Figure 6a). The results agreed with those of Akıllıoglu and Karakaya [48], who observed that the heat treatment produced an increase in the capacity of the ACE inhibition of green lentils.

The inhibitory activity of melanoidins can be partly associated with their metal chelating properties, as ACE is a Zn-dependent enzyme [49]. There is strong evidence that melanoidins can act as metal chelating agents, as their anionic nature permits them to chelate transition metals [50]. Other authors associated the ACE inhibitory activity with LMW Maillard compounds which bound to the melanoidin structure and compete for the active place of ACE [44,46]. The decrease of ACE observed at longer heating times would respond to the reduction in LMW compounds and production of HMW compounds. In our study, a decrease in ACE was not observed, although it may be necessary to evaluate longer times to observe this effect.

### 2.5. Digestible Starch (DS)

BS showed a negative linear behavior with respect to the temperature and time during the baking process (Figure 6b), finding the minimum values of bioavailable starch at the maximum baking temperatures (210 °C). Increasing the intensity of the baking temperature can cause a fast evaporation of water from the crust, owing to its high surface temperature impairing the full gelatinization and therefore digestibility of the starch [23]. Foster-Powell et al. [51] have described that different elements can present oscillations in the GI due to factors such as when the food is baked, increases in the GI, or the retrogradation process that the paste undergoes when chilled or stored.

### 2.6. Scanning Electron Microscopy (SEM)

**Figure 7:**
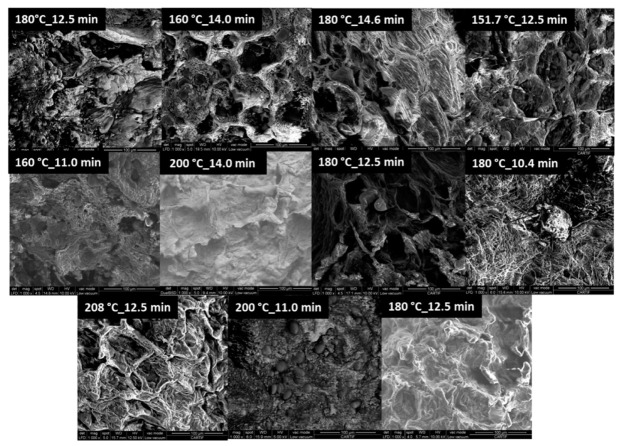
Scanning Electron Microscopy (SEM) for the snacks processed following the temperature and time RSM design.

The microstructure of the pulse snacks, as affected by the different baking conditions used, is shown in Figure 7. The SEM micrographs show structures in the interior of the samples related to the gas retention and coalescence of gas bubbles; these structures seem less defined and less regularly shaped with increasing processing temperatures. The samples treated at low and intermediate temperatures show more porous and lighter structures, which may result in decreased hardness and improved masticability and textural properties. Other authors have shown similar structures in gluten-free rice-based breads, but they are less regular and of a larger size [52]. The addition of pulse flour to the formulation, as is the case of the case of Narbon bean snacks produced, may result in improved gas retention and structure regularity, possibly due to the increased protein fraction and changes in the viscoelastic behavior of the dough throughout baking. On the other hand, the starch granule integrity was apparently higher in those samples baked at higher temperatures, resulting in a similar appearance to unbaked doughs [52], which may be related to a faster drying rate and limited gelatinization extent [23].

### 2.7. Colorimetry

**Figure 8:**
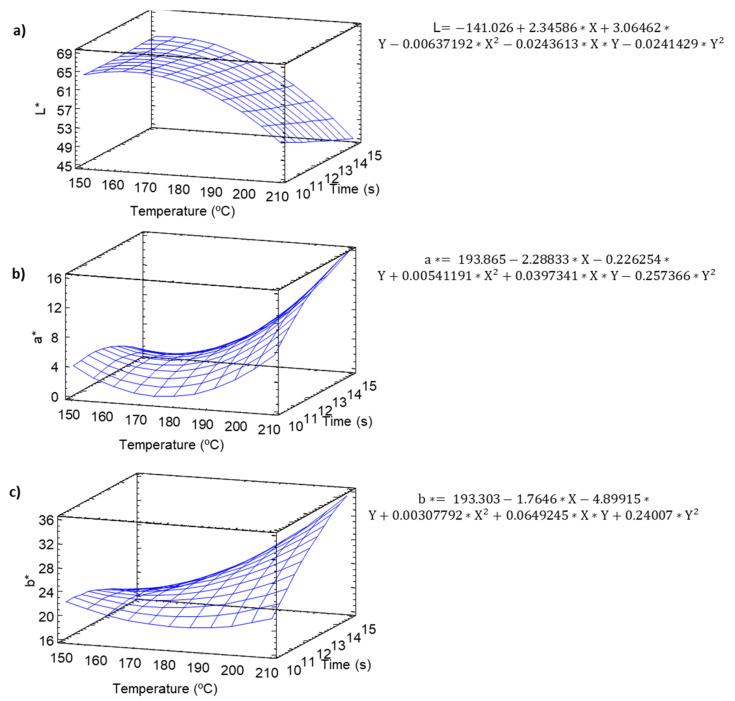
Color variation in the snacks. Equations and modeled variation in the effect of temperature and time on the CIELab parameters: (**a**) luminosity, (**b**) a* (green-red), (**c**) b* (blue-yellow).

Changes in color were analyzed in the study. A significant effect of temperature–time in this parameter (Supplementary Image S1, Figure 8) was observed. The luminosity decreased significantly during baking. The minimum values of luminosity were observed at (160 °C) and the maximum at (210 °C).

a* (Figure 8b) showed a positive quadratic exponential behavior, very similar to the behavior of b* (Figure 8c), finding the maximum values at high temperatures and maximum baking times of 210 °C and 14 min, which would be associated with a greater baking–that is, greater roasting. While at intermediate temperatures (180 °C) and minimum times (10 min), the impact on the color was much lower (Supplementary Image S1).

A principal component analysis (PCA) was performed (Supplementary Figure S2) in order evaluate if there was a relationship between color increase (melanoidins production) and antioxidant activity. The results showed that antioxidant activity was highly associated to increases in the a* value and decreases in luminosity.

### 2.8. Sensory Analysis

**Figure 9:**
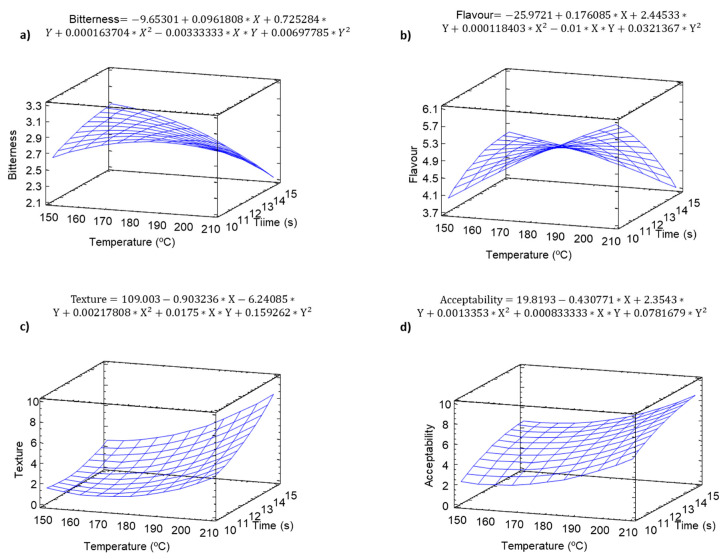
Sensory analysis of the snacks. Equations and modeled variations in the effect of temperature and time on (**a**) the bitterness, (**b**) flavor, (**c**) texture, (**d**) acceptability.

A sensory analysis was carried in order to evaluate the effect of temperature and time on the organoleptic properties of pulse snacks. The flavor and bitterness (Figure 8a,b) increased significantly (*p* ≤ 0.05) during baking, with maximum values at low baking times, which could be associated with the generation of melanoidns of LMW, since those effects are significantly reduced at longer baking times, where probably part of the LMW melanoidins are polymerized to HMW (*p* ≤ 0.05). There was a significant (*p* ≤ 0.05) increment in the acceptability of the snacks with temperature and time (Figure 9). The maximum acceptability values were observed at high temperature and time, conditions where texture values (Figure 8c), should better textural properties.

### 2.9. Simultaneous Optimization by the Desirability Function Approach

**Figure 10:**
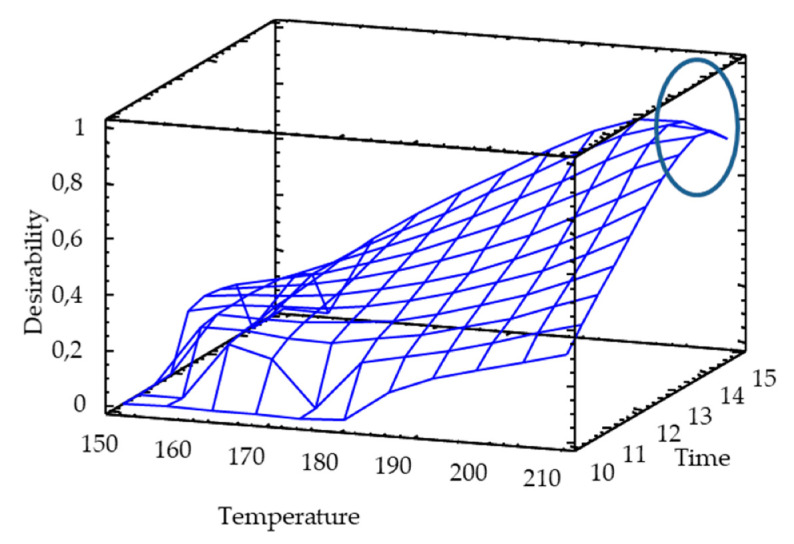
Simultaneous optimization by the desirability function approach.

The level of difficulty of multi-response optimization increases when the optimal areas of the responses are too distant and do not cross. It is therefore necessary to find an optimal compromise between all the significant responses. Thus, for the two-sided transformation of the responses to an individual desirability scale where they increased linearly from 0 to 1, significant variables such as antioxidant markers, humidity, and water activity were used. In this case (Figure 10), the antioxidant and antihypertensive properties were distant from the humidity and water activity. The desirability function showed than maximum temperature of 210 °C and time of 14 min were the optimum conditions to produce a pulse-based snack with a high antioxidant activity and quality.

## 3. Materials and Methods

### 3.1. Chemicals

α-Amylase from porcine pancreas (EC 3.2.1.1), angiotensin I-converting enzyme (ACE), 2,2′-Azinobis 3-ethylbenzothiazoline-6-sulfonic acid (ABTS), 2,2′-diazobis-(2-aminodinopropane)-dihydrochloride (AAPH), 2,2-diphenyl-1-picrylhydrazyl (DPPH), Folin-Ciocalteu (FC) reagent, gallic acid (GA), 6-hydroxy-2,5,7,8-tetramethyl-2-carboxylic acid (Trolox), o-aminobenzoylglycine (Abz-Gly), o-aminobenzoylglycyl-p-nitro-L-phenylalanyl-L-proline (Abz-Gly-Phe(NO2)-Pro), and sodium carboxymethylcellulose (CMC) were obtained from Sigma-Aldrich, Co. (St. Louis, MO, USA). Amyloglucosidase (EC 3.2.1.3) and the glucose oxidase-peroxidase (GOPOD) kit were purchased from Megazyme International Ireland (Wicklow, Ireland).

### 3.2. Grain Sample

*Vicia narbonensis* obtained from experimental fields of the Agricultural Technological Institute of Castilla y León (ITACyL, Valladolid, Spain) was used for the study. Seeds from cultivar ZV-145, with 1.74 g/100 g of GEC content, 10.5 g/100 g of humidity, and 31.2 g/100 g of protein, were used for the study. The grains were milled with a laboratory mill (Model 3100, Perten Instruments, Hägersten, Sweden), sifted (*Φ* = 0.5 mm), sealed in polyethylene bags under a vacuum, and stored at −20 °C until analysis. Rice flour was kindly provided by Emilio Esteban S.L. (Valladolid, Spain). Other baking ingredients, including baker’s yeast and salt, were obtained from a local market.

### 3.3. Snack Formulation

A gluten-free cracker based on a rice-Narbon bean-flour blend was formulated. During preliminary baking trials to define the maximum acceptable levels of Narbon bean flour, crackers were prepared containing 60% rice flour and 40% Narbon bean flour [9]. Dough samples were prepared by mixing 100 g of flour, 2 g of carboxy methyl cellulose (CMC), and 2 g baker’s yeast (pre-activated in 60 mL water). Additional water was added as required to obtain easy to handle doughs. After mixing all the ingredients for 5 min, the doughs were allowed to rest at room temperature (RT) for 10 min and subsequently incubated for 40 min at 37 °C to allow the fermentation process to take place. The snacks were baked in a forced-air convection oven at 180 °C for 12.5 min. The samples were cooled down at RT for 1 h before being placed into sealed polyethylene/plastic bags until further analysis.

### 3.4. Experimental Design and Data Modeling by Response Surface Methodology

A response surface methodology (RSM) with a face-centered rotational composite experimental design built with two levels of (−1 and +1) factorial design points and axial and center points was employed. A total of 12 experimental runs, including three center point and “star” points to estimate curvature, were conducted. The design was replicated, and the order of the experiments was randomized to protect against the effects of lurking variables (Table 2).

The combination of the two factors (baking temperature and time) was measured through the quality and bioactive parameters, selected as dependent factors in order to predict the optimal conditions to extend the shelf-life of the product with optimal healthy properties. The design was used to explore the quadratic response surfaces, and the polynomial equations were generated by the experimental design. RSM is based on the application of the multivariate design of experiments (DOE), followed by mathematical modeling and multiple response optimization using a desirability (D) function.

### 3.5. Proximal Characterization

The moisture content was analyzed gravimetrically using a drying treatment on the samples at 100 °C for 24 h. The fat content was measured using petroleum ether 40–60 °C for over 4 h of extraction, and the increase was gravimetrically determined. The nitrogen content was determined by the 968.06 AOAC method [53], and protein was calculated from the nitrogen content using the conversion factor of 6.25. The ash content was determined following the 923.03 AOAC method [53]. The total dietary fiber was determined with an enzymatic method, 985.29 AOAC [53]. All the parameters were expressed as grams per 100 g of sample.

### 3.6. Water Activity (a_w_)

The water activity was measured with an Aqualab 4TE water activity meter (Decagon Devices Inc., Pullman, WA, USA).

### 3.7. Phenolic Characterization and Content

#### 3.7.1. Total Phenolic Content (TPC)

The TPs were measured using the Folin-Ciocalteu reagent, according to Slinkard and Singleton [54], with modifications [55]. The absorbance was measured at 765 nm with a microplate reader (Fluostar Omega, BMG, Ortenberg, Germany). The results were expressed as the mg Gallic Acid Equivalent (GAE)/100 of sample on a dry weight basis (dw).

#### 3.7.2. Characterization of Free Phenolic Profile

For phenolic analysis, the samples were previously freeze dried (LyoQuest, Telstar, Terrassa, Spain). After drying, the samples (0.5 g) were extracted with 80% methanol by shaking for 30 min in an incubating mini shaker, then put in an ultrasonic bath for 30 min and further centrifuged at 6700 g for 5 min. The supernatants were collected and the residue was submitted to the same process twice. The three supernatants were combined and concentrated under reduced pressure to dryness. The residue was dissolved in 0.5 mL of formic acid 0.1%: acetonitrile (70:30, *v*/*v*), sonicated twice for 5 s, centrifuged (10.000 g, 2 min), and analyzed by HPLC using double online detection by diode array spectrophotometry and mass spectrometry (MS). A Hewlett-Packard 1200 chromatograph (Hewlett-Packard 1200, Agilent Technologies, Santa Clara, CA, USA) provided with a binary pump and a diode array detector (DAD) coupled with an HP ChemStation (rev. A.05.04) data processing station was used. The system was connected via the cell outlet to an MS detector API 3200 Qtrap (Applied Biosystems, Darmstadt, Germany) that was controlled by Analyst 5.1 software. The separation was achieved on an Agilent Poroshell 120 EC-C18, 2.7 μm (4.6 × 150 mm) column thermostated at 35 °C. The solvents were (A) 0.1% formic acid and (B) acetonitrile. The elution gradient established was isocratic 15% B for 5 min, 15–20% B over 5 min, 20–35% B over 10 min, 35–50% B over 10 min, 50–60% B over 5 min, isocratic 60% B for 5 min, and the re-equilibration of the column to the initial solvent conditions. The flow rate was 0.5 mL/min. Double online detection was carried out in the DAD at 280, 330, and 360 nm as the preferred wavelengths and in the MS operated in the negative ion mode. The spectra were recorded between *m*/*z* 100 and *m*/*z* 1000. Zero grade air served as the nebulizer gas (30 psi) and as turbo gas (400 °C) for solvent drying (40 psi). Nitrogen served as the curtain (20 psi) and collision gas (medium). Both the quadrupoles were set at unit resolution, and EMS and EPI analyses were also performed. The EMS parameters were: ion spray voltage −4500 V, DP −50 V, EP −6 V, CE −10 V, and cell exit potential (CXP) −3 V. The EPI settings were: DP −50 V, EP −6 V, CE −30 V, and CES 10 V. The individual phenolic compounds were tentatively identified from their UV and mass spectra and compared with the data reported in the literature.

### 3.8. Total Antioxidant Capacity (TAC)

The TAC was measured in the methanolic extracts using the 2,2-Diphenyl-1-picrylhydrazyl (DPPH) radical scavenging activity, the Oxygen Radical Absorbance Capacity (ORAC), and 2,2′-Azinobis-(3-ethylbenzothiazoline-6-sulfonate (ABTS) methods. In addition, the DPPH and ABTS methods on solid samples without previous extraction (Q-DPPH and Q-ABTS) were carried out. The reagents were provided by Sigma-Aldrich (Madrid, Spain).

#### 3.8.1. Extracts Preparation

One gram of each finely ground sample was extracted with 10 mL of methanol:water (1:1, *v*/*v*; acidified to pH = 2 with 0.1M HCl) in a temperature-controlled orbital shaker (25 °C, 250 rpm, 1 h). After centrifugation (25 °C, 3800 g, 10 min), the supernatant was collected, filtered (Whatman paper Nº 1), adjusted to 25 mL with the extracting solvent and stored at −80 °C until further analysis.

#### 3.8.2. DPPH (Classical and Quencher) Assays

The antioxidant activity of the extracts against the DPPH radical was estimated according to the procedure described by Brand-Williams et al. [56], with modifications. An amount of 25 µL of extracts was mixed with 100 µL of MilliQ water and 125 µL of DPPH working solution (100 µM using methanol as solvent) in a 96-well microplate. The absorbance at 515 nm was recorded for 30 min in a microplate reader (Fluostar Omega, BMG, Ortenberg, Germany). The results were corrected for moisture and expressed as mg Trolox Eq./100 g of the sample on a dry weight basis (dw).

The DPPH-Q method was evaluated following the procedure described by Serpen et al. [57], with modifications. Ten milligrams of powdered samples (particle size below 300 µm) were mixed with 1.5 mL of 60 µM DPPH methanolic solution. After incubation at 700 rpm for 30 min (Thermomixer Compact, Eppendorf AG, Hamburg, Germany), the samples were centrifuged at 14,000× *g* for 2 min and the absorbance measured at 515 nm in a microplate reader. The results were corrected for moisture and expressed as the mg Trolox Eq./100 g of sample on a dry weight basis (dw).

#### 3.8.3. ORAC Assay

The procedure was based on a previously reported method with slight modifications [58]. The standard curve of Trolox (15–240 mM) and the samples were diluted in phosphate buffer (10 mM, pH 7.4). A volume of 150 μL of fluorescein was placed in 96-well black polystyrene plates, and 25 μL of Trolox standard, the sample, or phosphate buffer as a blank were added, all in duplicates. The samples, standards, and blanks were incubated with fluorescein at 37 °C for 3 min before 2,2′-azobis (2-methyl propionamidine) dihydrochloride solution was added to initiate the oxidation reaction. The fluorescence was monitored over 35 min with a microplate reader, using 485 nm excitation and 528 nm emission filters. The results were calculated using the areas under the fluorescein decay curves, between the blank and the sample, and expressed as the mg Trolox Eq./100 g of sample on a dry weight basis (dw).

#### 3.8.4. ABTS (Classical and Q-Versions) Assay

The antioxidant capacity against the diammonium salt of ABTS radical was evaluated following the method first described by Miller and Rice-Evans [59], as modified by Martin-Diana et al. [55]. The absorbance was measured at 730 nm with a microplate reader. The results were corrected for moisture and expressed as mg Trolox Eq./100 g of sample on a dry weight basis (dw).

The ABTS-Q method described by Serpen et al. [57], as modified in Martin-Diana et al. [55], was used to evaluate the direct antioxidant capacity of the wheat bran samples. The results were corrected for moisture and mg Trolox Eq./100 g of sample on a dry weight basis (dw).

### 3.9. Angiotensin I-converting Enzyme (ACE) Inhibitory Activity

The ACE inhibitory activity was measured using a fluorometric microtitre assay, as described by Sentandreu and Toldrá [60]. Briefly, an ACE solution of 15 mU/mL was prepared by diluting the enzyme solution appropriately with enzyme buffer (150 mM Tris-base buffer, pH = 8.3). The same aqueous methanolic extracts obtained for TAC determinations were diluted with the extracting solvent to test different concentrations of each sample. The assay was run continuously for 30 min at 37 °C and fluorescence from the release of Abz-Gly was quantified at time zero and time 30, using a microplate reader, with excitation and emission wavelengths of 360 nm and 400 nm, respectively. The ACE inhibition (%) for each reaction was determined, and the concentration of sample in the final assay volume that inhibited 50% of the ACE activity (IC_50_ values) was calculated by plotting the sample concentration (g/L) versus the % of ACE inhibition. The final results for the IC_50_ values were corrected for moisture and expressed as the gram of sample per liter of medium present during the reaction (g/L). The antihypertensive drug captopril was used as a reference for the ACE inhibitor to ensure the assay reproducibility. The experiments were carried out as independent duplicates assayed in triplicate.

### 3.10. Determination of Available Starch (BS)

The available starch in the crackers was measured following the protocol described in the total starch assay kit of Megazyme (K-TSTA 08/16). The available carbohydrate content in the samples was estimated using a D-glucose standard solution (1.0 mg/mL) as a reference, and the available starch was calculated as 0.9× glucose (mg). The final results were corrected for sample moisture and expressed as the % (*w*/*w*) of dry matter (DM).

### 3.11. Scanning Electron Microscopy (SEM)

SEM was evaluated using a scanning electronic microscope (FEI QUANTA 200, Graz, Austria). Images were taken using 150× and 1000× magnifications for surface and section. Voltages between 5 and 15 kV were used depending on the detector and the topography of the sample and the spot sizes.

### 3.12. Colorimetric Analysis

Lightness (L*), redness (a*), and yellowness (b*) were measured using a colorimeter (Konica Minolta, CM-2600d, Osaka, Japan). The illuminant was D65 (color temperature of 6504 K). The colorimeter was standardized using a light trap and a white calibration plate. Measurements were taken for the samples at 10 different points.

### 3.13. Sensory Analysis

A panel of eight panelists with previous experience in sensory analysis, aged between 20 and 48 years old, were recruited from ITACyL Staff. Training was carried out in common sessions, using commercial cracker products in order to establish consensus on the descriptors. The color intensity, texture (crispness), aroma, and acceptability descriptors were evaluated using a 9-point scale, with 1 = minimum and 10 = maximum intensity. Each judge was presented with a complete set of samples with random 3-digit codes for evaluation, generating one vector of multiple dependent data, as shown by Carabante and Prinyawiwatkul [61].

### 3.14. Statistical Analysis

In RSM, the systems of equations were solved using least squares (LS) method, which assumes that random normal errors are identically distributed with a zero mean and a common unknown variance and are independent of each other. The descriptive capability of the model was quantified by the coefficient of determination (R2). Factors (VIFs) were evaluated, since they provide an index that measures how much the variance (the square of the estimate’s standard deviation) of an estimated regression coefficient is increased because of collinearity. A second-degree model was used to evaluate the responses. Statgraphic Centurion XVI was used for carrying out all the statistical analyses.

## 4. Conclusions

The optimal baking parameters (temperature and time) for pulse (Narvon) bean-containing snacks were obtained, conditioned to maximize the bioactivity and nutritional profile in order to the increase product shelf-life without compromising on the sensorial attributes. The results showed that the processing of the snack benefits from a temperature of 200 °C and 14 min time, among the range of conditions studied. The increase in the temperature also enhanced the bioactive properties of the snack, probably associated with the Maillard reaction products, which increased the scavenging activity and antihypertensive activity of the final product.

## Figures and Tables

**Table 1:** Proximal composition of the snacks, and equations and modeled variations for the effect of temperature and time on protein, carbohydrates, fiber, and ash.

Snack	Temperature (°C)	Time (Minutes)	Fat (%)	Protein (%)	Carbohydrates (%)	Fiber (%)	Ash (%)
1	180	12.5	1.23	14.84	76.86	11.1	3.2
2	160	14	1.17	14.96	77.03	10.7	3.22
3	180	14.62	1.79	15.01	76.42	10.8	3.2
4	151.72	12.5	1.32	15.14	77.29	10.2	3.27
5	160	11	1.27	14.91	77.19	10.8	3.26
6	200	14	1.47	15.03	76.93	11.1	3.2
7	180	12.5	1.44	14.94	76.79	11.3	3.21
8	180	10.38	1.45	15.06	76.7	10.9	3.2
9	208.28	12.5	1.26	14.86	77.07	11.8	3.16
10	200	11	1.28	14.89	76.87	10.6	3.17
11	180	12.5	1.12	14.94	76.9	10.5	3.2

**Table 2:** Response surface methodology (RSM) with a face-centered rotational composite experimental design.

Snack	Temperature (°C)	Time (minutes)
1	180.0	12.5
2	160.0	14.0
3	180.0	14.6
4	151.7	12.5
5	160.0	11.0
6	200.0	14.0
7	180.0	12.5
8	180.0	10.4
9	208.3	12.5
10	200.0	11.0
11	180.0	12.5

## Supplementary Materials

The following are available online, Figure S1: Pareto chart for Total polyphenol content (TPC), Figure S2: Principal component analyses for baking process respect to temperature and time, Table S1: Total polyphenol content (TPC) statistic, Image S1: Pulse-based crackers design.
