# Supplementary material for: Baking Optimization as a Strategy to Extend Shelf-Life through the Enhanced Quality and Bioactive Properties of Pulse-Based Snacks
Source: Molecules. 2020 Aug 14;25(16):3716. doi: 10.3390/molecules25163716 (PMC7463836; doi:10.3390/molecules25163716)

Supplementary Figure 1

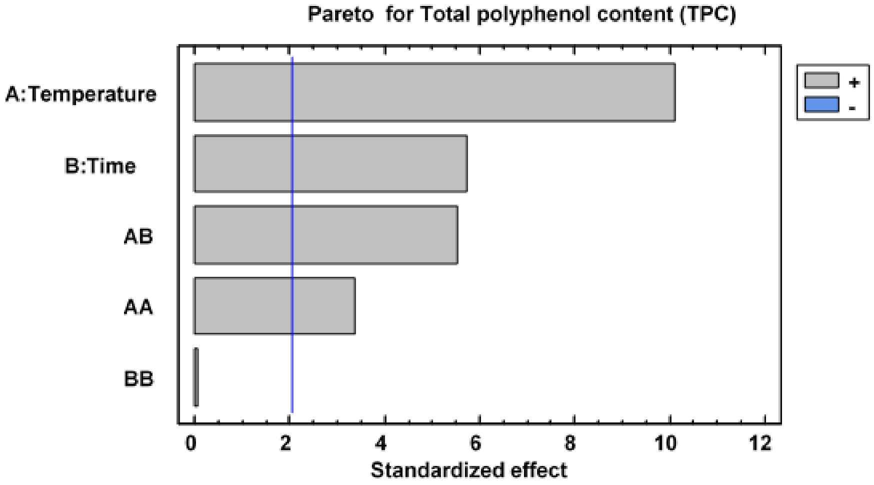

Supplementary Table 1

Variable	*Sum of squares*	*Gl*	*Medium squares*	*Razón-F*	*value-P*
A:Temperature	16316.4	1	16316.4	102.41	0.0000
B:Time	5238.27	1	5238.27	32.88	0.0000
AA	1821.3	1	1821.3	11.43	0.0023
AB	4876.3	1	4876.3	30.61	0.0000
BB	0.781876	1	0.781876	0.00	0.9447
Bloq	0,504041	1	0.504041	0.00	0.9556
Error	4142.54	26	159.328		
Total (corr.)	32538.9	32			

R-square = 87.269 %

Supplementary Figure 2

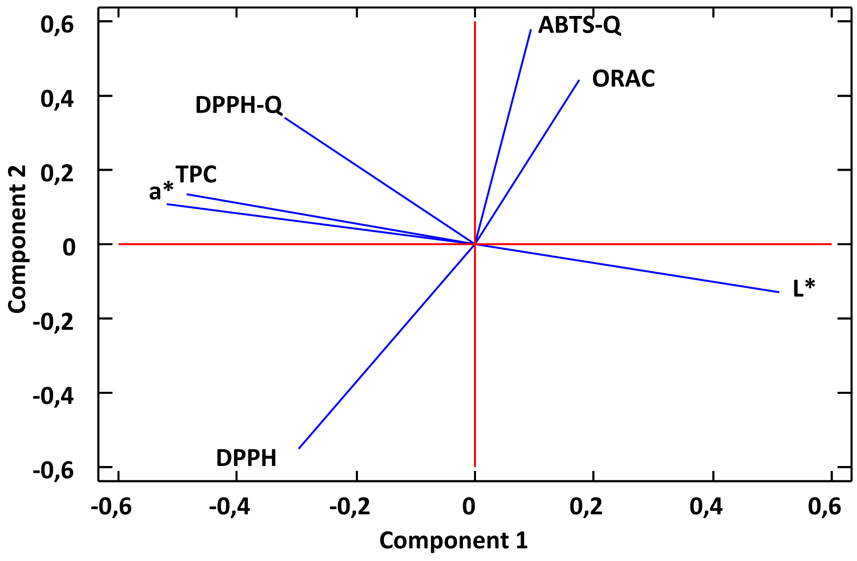

Supplementary Image 1

1

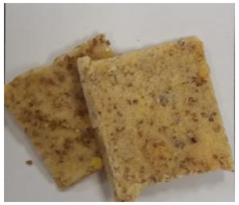

2

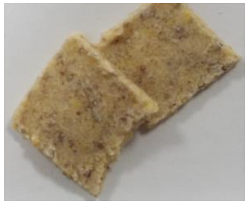

3

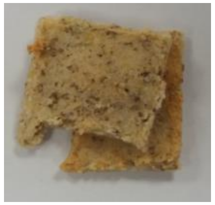

4

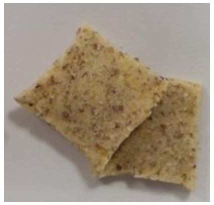

5

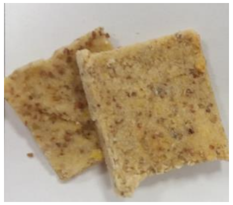

6

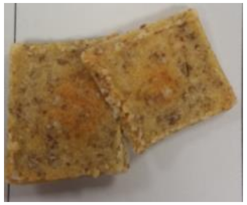

7

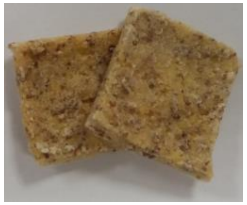

8

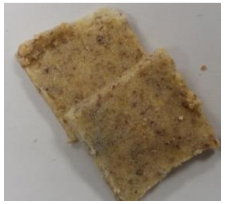

9

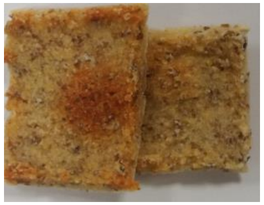

10

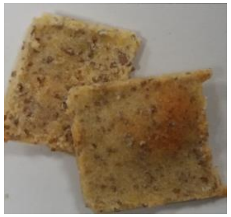

11

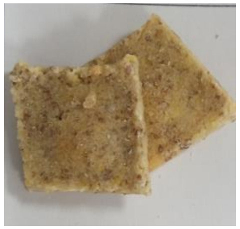

Supplement: Supplementary file 1 [file molecules-25-03716-s001.pdf]
